# The effect of health on refugees’ labor market integration: Evidence from a natural experiment in Germany

**DOI:** 10.1371/journal.pone.0346936

**Published:** 2026-04-20

**Authors:** Laura Goßner, Philipp Jaschke, Yuliya Kosyakova

**Affiliations:** 1 Institute for Employment Research (IAB) of the Federal Employment Agency (BA), Nuremberg, Germany; 2 Otto-Friedrich-University Bamberg, Bamberg, Germany; Yunnan University, CHINA

## Abstract

This paper analyzes the role of health for refugees’ integration into host countries’ labor markets. We exploit the quasi-random dispersal policies of refugees across regions in Germany to analyze the causal effect of health on employment. Based on regional and temporal heterogeneity in a policy adoption that provided earlier access to healthcare services through electronic health cards (eHCs), combined with the regional availability of healthcare services and pre-migration health status, we construct instrumental variables (IVs) providing plausibly exogenous variation in refugees’ post-arrival health status. Our results reveal that favorable physical health (PCS) improves refugees’ employment probability. Favorable mental health (MCS) increases only females’ employment rates, although this effect must be scrutinized due to weak instruments. Regarding potential mechanisms, we provide evidence that better health increases language course participation and German language proficiency for female refugees.

## 1. Introduction

With rising numbers of individuals seeking safety across borders [1], the labor market integration of refugees has become a central concern for policymakers, the public, and researchers alike. An extensive body of literature is examining factors that facilitate or hinder refugees’ integration into host country labor markets (see Kosyakova and Kogan [2] for an overview). Individual-level resources such as human capital (e.g., language skills, education, work experience) and social capital have been shown to play a central role in promoting successful labor market integration [e.g., 3–5]. However, one key dimension remains underexplored: the role of refugees’ physical and mental health – although it has been a focus of research across various disciplines in its own right [6–8] – in shaping integration outcomes.

Studying this link is critical because both poor health and delayed labor market integration are widespread among refugee populations. In Germany, nine years after arrival, about 64 percent of working-age refugees are employed – only six percentage points below the average in the general population [9]. However, this convergence takes considerable time, and many refugees remain unemployed or underemployed for years after arrival. Similar patterns have been identified across a range of western high-income countries [10–12]. At the same time, health problems are highly prevalent: 28 percent of recently arrived refugees suffer from post-traumatic stress disorder (PTSD) [13], and 41 percent report significant psychological distress [8]. Physical health issues are also widespread (see Hadgkiss and Renzaho [14] for an overview); for example, 60 percent of asylum seekers in the Netherlands report poor health, with nearly half affected by one or more chronic conditions [15]. These health burdens can delay labor market entry, hinder participation, and reinforce existing structural barriers to integration.

Germany provides a particularly relevant case for studying the role of health in refugee labor market integration. Since 2015, Germany has received more than 2.3 million asylum applications – the highest number in Europe during this period [16]. As a result, it has become a central site for integration research, with substantial investments in housing, language training, and labor market access. Despite these efforts, refugee integration trajectories are marked by prolonged employment gaps, strong gender disparities, and challenges in the recognition of pre-migration qualifications [17]. At the same time, recent evidence suggests that refugees’ sense of being welcome in Germany has declined over time, while fears of xenophobia have increased [18,19]. In addition, although refugees tend to report relatively good physical health – partly due to their younger age structure – their mental health outcomes are significantly worse than those of the general population, particularly among older refugees (aged 45 and above) [20]. Furthermore, Germany’s federal structure leads to substantial regional variation in access to healthcare and integration services, making it a suitable setting for studying the causal effects of health-related policy exposure [21].

In the general population, a substantial literature highlights the bi-directional relationship between health and employment. The causation hypothesis suggests that employment has protective effects on health, while the selection hypothesis argues that poor health itself reduces employment chances [22,23]. Empirical research supports both mechanisms: employment improves mental health outcomes and reduces depression [24,25], while individuals with health impairments face increased risks of job loss and prolonged unemployment [26,27]. Few studies that have tried to disentangle both effects, concluded that both mechanisms work simultaneously [22,23,28]. However, reverse causality and endogeneity make it methodologically challenging to empirically identify effects in both directions, particularly in vulnerable subpopulations such as refugees.

While these mechanisms are well established in the general population, they may operate differently among refugees, who face unique vulnerabilities – including pre- and post-migration stressors, legal uncertainties, and structural disadvantages in host societies. Refugees often flee zones affected by conflict, violence, or human rights violations [1] and are exposed to traumatic experiences such as natural disasters, physical or sexual assault, captivity, torture, severe human suffering or shipwrecks on their way to safer countries [13,29]. Upon arrival, they frequently encounter post-migration stressors such as legal uncertainty, discrimination, limited access to healthcare, and precarious living conditions [6,30,31]. These compounded stressors contribute to the high prevalence of both mental health disorders and physical health issues [6,14,15,32].

Despite this evidence, the consequences of poor health for refugees’ labor market integration remain under-researched [33]. Overall, most studies point to a positive association between mental health and employment (see [34] for the Netherlands, [35] for Canada, [36] for Australia, [8] for Germany, [37] for Switzerland, and [38] for the UK), though a few report no effect (see [39 for Germany, 40 for Sweden]) – possibly due to differences in sample selection, data quality, or national contexts. Evidence on physical health is even scarcer, though some studies have documented positive associations with employment outcomes [38,41]. As a recent review by Lai, Due and Ziersch [33] concludes, empirical studies remain largely descriptive, and causal evidence on the role of both mental and physical health in shaping refugees’ labor market participation is still lacking.

We address these gaps by providing causal evidence on how both mental and physical health influence labor market participation among refugees in Germany. To this end, we exploit a natural experiment stemming from policy-induced variation in access to healthcare services across time and regions in Germany. Specifically, we instrument refugees’ health status using plausibly exogenous differences in healthcare eligibility rules, the local availability of medical services, and individual-level pre-migration health indicators. This identification strategy allows us to isolate the impact of health on employment outcomes while addressing concerns of reverse causality and endogeneity. We combine this with rich data from the longitudinal and representative IAB-BAMF-SOEP survey of refugees in Germany [42]. To explore possible effect heterogeneity, we estimate models separately for men and women – a distinction motivated by research showing that refugee women face distinct health risks, including exposure to sexual violence [43,44], reproductive health challenges [43,45] and elevated rates of depression or anxiety [46–48].

Our instrumental variable (IV) results reveal that favorable physical health significantly increases refugees’ employment probability. Furthermore, favorable mental health appears to improve employment rates only among women, though this effect is less robust due to weak instruments. In exploring underlying mechanisms, we find that better health increases language course participation and German language proficiency, mostly among women – pointing to indirect pathways through which health may influence integration.

The remainder of the paper is structured as follows: Section 2 describes the policy context and the natural experiment regarding healthcare access. In section 3, we present the data, variables and our analytical strategy. Section 4 outlines the results, while Section 5 provides discussion and conclusions.

## 2. Policy context

As variation in healthcare access policies – linked to refugee allocation procedures across federal states – serves as a natural experiment in our study and is essential for our identification strategy, this section outlines the policy context in more detail.

In Germany – similarly to many other refugee destination countries [7] – refugees encounter significant hurdles in accessing healthcare services. Upon arrival in Germany, their access is regulated by the German Asylum-Seekers Benefits Act (*Asylbewerberleistungsgesetz*, AsylbLG), established in 1993. This legal framework governs social benefits for asylum seekers whose application is still pending, as well as for rejected asylum seekers with an obligation to leave or a tolerated status (*Geduldete*).

The AsylbLG limits healthcare coverage primarily to acute illnesses, conditions of pain, care for pregnant women, vaccinations, and medically necessary preventive examinations (para. 4). Additional treatments may be granted on a case-by-case basis (para. 6). Full access to regular healthcare services is granted only after a positive asylum decision or after a minimum duration of stay in Germany (para. 2). This required duration has been subject to change in previous reforms: it was initially set at 12 months in 1993; increased to 36 and 48 months in 1997 and 2007; reduced to 15 months in 2015; and again raised to 18 months in 2019. In early 2024, the required duration period was increased to 36 months [49].

Focusing on refugees who arrived in Germany between 2013 and 2019, Biddle [50] shows that most individuals experienced restricted healthcare access for slightly over a year before becoming eligible for full coverage. Two-thirds reached eligibility through the maximum legal waiting time, while around one third gained access sooner through a positive asylum decision.

For those covered under AsylbLG, two different administrative systems govern access to healthcare services [51], depending on the federal state. In seven of Germany’s sixteen federal states, a treatment voucher system remains in place. Under this system, refugees must request a voucher from the local foreigners’ authority or social assistance office before visiting a doctor – except in emergencies. The voucher is issued only if the treatment falls within AsylbLG provisions [51]. This process has been widely criticized for its bureaucratic complexity, delays in treatment, and the fact that treatment decisions are made by non-medical personnel [21,51,52]. These shortcomings often lead to inadequate healthcare provision [32,51] and ultimately worse health outcomes [21].

In contrast, an increasing number of states have adopted a more inclusive approach by introducing electronic health cards (eHC). These are issued through agreements with health insurance providers and allow refugees to access medical care without prior approval. While the eHC initially mirrors AsylbLG coverage, it often approximates the benefits of regular health insurance in practice [53]. The adoption of eHCs began in 2005, and has since expanded across several federal states, though with significant variation in timing and geographic coverage. As of 2024, seven states have adopted the eHC system comprehensively, and three others had adopted it partially at the municipal or district level [50]. A detailed overview of the regional coverage and implementation timeline is provided in Supplementary Table S1 in S1 File. Jaschke and Kosyakova [21] show that the introduction of eHCs led to measurable improvements in both mental well-being and subjective health among eligible refugees.

The institutional variation in healthcare access is exogenously assigned due to Germany’s refugee allocation system. Upon their arrival, refugees in Germany are quasi-randomly distributed across federal states (following the annually updated *Königsteiner* Schlüssel, which is based on tax-revenue and population numbers). In addition, approved refugees are subject to residence restrictions for up to three years [21], limiting their ability to relocate. As a result, access to healthcare systems – whether based on treatment vouchers or electronic health cards (eHCs) – is effectively assigned by chance. This quasi-random allocation creates conditions resembling a natural experiment, which we leverage to estimate the causal effect of healthcare access, operating through health, on refugees’ labor market outcomes.

Correspondingly, we use variation in eHCs eligibility as one of several instrumental variables for refugee health status. Specifically, we distinguish three groups:

- Individuals who were eligible for the eHC immediately upon their arrival due to being assigned to a district that has already adopted the eHC system;
- Individuals who gained eligibility before reaching maximum waiting time as defined in the AsylbLG (15 months for asylum seekers in our sample) – either due to early asylum approval or due to being in a region that implemented the eHC policy during that period;
- Individuals who became eligible for the eHC only after having reached the maximum waiting time of 15 months.

In sum, the staggered introduction of eHCs not only created institutional variation in healthcare access across regions, but also led to meaningful differences in health outcomes. Jaschke and Kosyakova [42] document that earlier access to eHCs significantly improved health among refugees. These improvements in health provide the critical link to our study, as we examine whether such gains in health translate into better labor market integration outcomes.

## 3. Data and method

### 3.1. Data and sample

For our empirical analysis we rely on data from the IAB-BAMF-SOEP survey of refugees, a longitudinal survey of refugees and their household members in Germany that is conducted since 2016 [42]. The sample is drawn from the Central Register of Foreigners (AZR) and covers individuals who arrived in Germany between 1st of January 2013 and 30th of June 2019 to seek protection for humanitarian reasons – irrespective of their legal status at the time of being drawn into the gross sample or being surveyed [54]. Interviews are conducted face-to-face with computer assistance (CAPI) and supported by interpreters if needed. Questionnaires are provided in seven languages (Arabic, English, Farsi/Dari, German, Kurmanji, Pashto, and Urdu).

We use the data version 36 covering survey years 2016–2019 [55], published and accessed by the authors on April 15, 2021 in fully anonymized form, with no information included that could identify individual participants. Our study did not require review by an ethics committee because the underlying data are secondary (retrospective) survey data that are publicly available for research purposes as part of the scientific use file (SuF) shared by the Research Data Center of the Socio-economic Panel (SOEP) upon contractual agreement. No additional (prospective) information was collected from respondents for our study other than that available to the general research community.

The survey includes more than 8,000 adults aged 18 or older who were interviewed at least once. We restrict our data to survey years until 2019 to rule out potential distortions due to the coronavirus pandemic. We further limit the analytical sample to refugees of working age (18–64 years), dropping those who arrived earlier than 2013 or have missing values in the most relevant variables (health outcomes, assigned district of residence, date of arrival, outcome and date of asylum decision). Finally, we keep individual observations with approved asylum applications, ensuring that all individuals in our sample are generally eligible for an eHC at the times of being surveyed, but have different preceding waiting periods. Following these restrictions, our main sample contains 3,454 persons (1,376 women and 2,078 men) contributing 5,041 person-year observations (1,935 women and 3,106 men).

### 3.2. Outcome variables

The labor market integration of refugees is assessed using the self-reported employment status at the time of the interview. Following the definition of the International Labour Organization, employment is defined as work performed in return for pay or profit [56]. We construct the outcome variable *employed* as a dummy variable, with one for respondents in full- or part-time employment, vocational education, internships, apprenticeships or marginal employment if they indicate gross monthly earnings above zero and coded ‘0’ otherwise. The unweighted mean employment rate in our sample is 17 percent (see Table 1).

**Table 1:** Summary Statistics of Main Variables.

Variable	Mean	SD	Min	Max	Median	N
*Outcome variables*						
Employed	0.17		0	1		5,041
German language proficiency (0 low – 12 high)	5.8	3.0	0	12	6.0	5,038
Language course (currently or previously)	0.81		0	1		4,992
*Measures of health*						
Mental Component Summary Scale (MCS)	48.8	11.3	4.6	77.9	49.7	5,041
Physical Component Summary Scale (PCS)	53.5	9.9	12.3	77.7	56.5	5,041
*Instruments*						
Satisfaction with health before migration (0 low – 10 high)	8.3	2.4	0	10	9	5,041
Months until eHC access: 0	0.08		0	1		5,041
1 - 8	0.45		0	1		5,041
9 - 14	0.26		0	1		5,041
15	0.22		0	1		5,041
General practitioner availability (distance in km)	9.5	5.5	2.8	33.6	8.2	5,041

*Source*: IAB-BAMF-SOEP Survey of Refugees, v36.

Additionally, we use German language proficiency and language course participation as outcome variables in section 4.3 to study potential mechanisms. Language proficiency is operationalized as an additive score of self-assessed speaking, writing and reading skills (each scaled from 0 `very poor’ to 4 `very good’). The mean score in our sample is 5.8. For language course participation, we code a dummy indicating whether refugees have been participating in a language course. These include integration courses provided by the Federal Office for Migration and Refugees (BAMF), ESF-BAMF vocational language courses that teach more specific German skills relevant to the intended occupation, courses provided in collaboration with the Federal Employment Agency or any other language courses. 81 percent of refugees in our sample have attended or are currently attending one of the above courses.

### 3.3. Measures of health

Mental and physical health are measured using the Mental and Physical Component Summary Scales (MCS and PCS) respectively. We compute the MCS and PCS based on the 12-item Short-Form version 2 questionnaire (SF-12v2), which forms a shortened version of the SF-36 short questionnaire on health-related quality of life [57].

In the IAB-BAMF-SOEP survey of refugees, the items were only surveyed for all respondents in even-numbered survey years. In odd-numbered years, only first-time respondents were polled. The included twelve items are condensed into eight distinct variables which capture physical functioning, role limitations due to physical or emotional problems, bodily pain, general health, vitality, social functioning and emotional well-being. We follow the procedure applied to the SOEP-Core survey as described by Andersen et al. [58] to construct the MCS and PCS scores. Both scales range from 0 to 100 with higher numbers indicating a better health status. The scales are normalized such that a value of 50 corresponds to the mean of the German population in the year 2004 and 10 points correspond to one standard deviation.

In the literature, the MCS has been shown to be a valid measure of mental health and suitable as a screening instrument for depression and anxiety disorders [59,60]. Similarly, the PCS’s validity has been tested in various settings in the context of different physical conditions [61,62]. However, SF-12 based measures of health have been shown to be only weakly cross-culturally comparable [63], which makes it important to control for further respondents’ characteristics (see the following section). Descriptive statistics of both MCS and PCS are presented in Table 1.

### 3.4. Covariates

All models control for individual- and contextual-level predictors of labor market integration in general and for refugees in particular. These include (1) *socio-demographics*: age, gender, partnership status, children in household and citizenship; (2) *human-capital characteristics*: pre-migration education, pre-migration German language skills, pre-migration employment status and illiteracy in their mother tongue; (3) *refugee-specific characteristics*: years since arrival, traumatic experience during escape, months of the asylum procedure, and post-migration stressors [64] such as type of accommodation, experience of origin-based discrimination, worries about prospects of staying in Germany and feeling welcome in Germany at arrival; (4) *survey-year fixed effects* to absorb any unobserved variation that affects all individuals in corresponding survey year (e.g., macroeconomic recessions); and (5) *district-level characteristics* of refugees’ assigned residence place by authorities (measured at baseline, i.e., in the year prior to their arrival): unemployment rate [65], population density, share of foreigners, share of refugees among foreigners [66] and the voting share of the right-wing party AfD in the federal elections in 2013 [67]. Note that months of the asylum procedure is collinear with months until eHC eligibility for refugees who have not been allocated to a district that implemented the reform providing immediate eHC access.

Detailed descriptive statistics are presented in supplementary Table S2 in S1 File. Almost two-thirds of the sample are men, 60 percent are younger than 36, and two-thirds of respondents reside with at least one child. The majority comes from Syria (69 percent), followed by Iraq (12 percent) and Afghanistan (8 percent), and most of the respondents have been living in Germany for between 1 and 4 years. Pre-migration education level is polarized, with almost a quarter exhibiting only primary or lower level of pre-migration education, and 21 percent having tertiary education. Five percent of the refugees are illiterate in their mother tongue (i.e., cannot read or write) and their level of German at the time of arrival was very low. Given lack of German skills at arrival, refugees without an eHC likely face barriers to accessing medical treatment when they must explain their symptoms to non-medically trained staff of social and immigration authorities to obtain treatment vouchers. Figures on communal accommodation, discrimination experiences and worries about prospects of staying in Germany suggest a substantial degree of post-migration stress among refugees.

### 3.5. Analytical approach

#### Ordinary least squares.

To estimate the association between health and employment, we begin with ordinary least squares (OLS) regressions. Standard errors are clustered at the person-level. We regress a dummy variable indicating employment of individual *i* having arrived in year *y* in district *d* in survey year *t* separately on MCS and PCS:

$${\text{1}\text{0}\text{0}[Employed]}_{i,y,d,t}={\beta_{\text{1}}\times health}_{i,t}+\beta_{2}\times {X_{1}}_{i,t}+\beta_{3}\times {X_{2}}_{i}+\beta_{4}{X_{3}}_{y-\text{1},d}+\gamma_{t},$$

where *health* denotes either MCS or PCS, $X_{1}$ and $X_{2}$ time-varying and time-constant confounding variables, respectively, $X_{3}$ district- and arrival year-specific variables, and $\gamma_{t}$ survey year fixed-effects. We run separate analyses for the whole sample, females, and males. In all regression tables, coefficients and standard errors are multiplied by 100 for readability and express the results in percentage points.

#### Instrumental variable (IV) approach.

Due to potential endogeneity and reverse causality, OLS estimates are likely biased. We therefore employ a two-stage least squares (2SLS) instrumental variable (IV) approach, leveraging plausibly exogenous variation in refugees’ health status. Instrumental variables have previously been used in other settings to overcome endogeneity of individuals’ health status, e.g., mental health in Lebenbaum et al. [68] and disability in Trani et al. [69].

We instrument refugees’ health (MCS and PCS) with the following three instruments. First, individual-level *months until eHC eligibility*, is coded into four categories: (i) 0 months, (ii) 1–8 months, (iii) 9–14 months and (iv) 15 months. Following Table 1, 8 percent of refugees were eligible to the eHC immediately after arrival, whereas 45 percent had to wait up to 8 months, 26 percent between 9–14 months and the remaining 22 percent 15 months. Second, regional *availability of medical services* is measured with a variable indicating the mean distance to the nearest general practitioner (GP) among district residents [66]. The average distance lies at 9.5 kilometers and is, hence, difficult to overcome without sufficient public or private transport availability. Third, individual-level *pre-migration health* is measured using self-reported satisfaction with health before migration, measured on a scale from 0 (low) to 10 (high). With 8.3, respondents score, on average, high on pre-migration health satisfaction. We also include interaction terms between these instruments to increase variation and improve identification.

For 2SLS analysis to provide consistent estimates, instruments are required to be ‘relevant’, i.e., correlated with the endogenous variable and to be ‘exogenous’, i.e., have no direct effect on the outcome conditional on the endogenous and exogenous variables, which is referred to as the exclusion restriction [70,71]. We argue that for all three instruments the requirement of *relevance* is satisfied given previous literature: First, for the same setting of refugees in Germany, Jaschke and Kosyakova [21] provided evidence that earlier accessibility of medical services through eHCs improves health outcomes of refugees. Second, several studies revealed the importance of medical services’ regional availability for health outcomes [72,73]. Refugees benefit, in particular, since they rarely possess a private car to reach a distant GP [74]. And third, past health status has been shown to be highly predictive for current health status in general populations [75], for refugees [76,77] and even across generations [78]. This allows us to use pre-migration health status as a predictor for current health outcomes in Germany. Similar instruments have been used in related work (e.g., Schuss [79] instruments current language skills with pre-migration proficiency).

The *exclusion restriction* of the instruments cannot be tested empirically, but we argue that it is plausibly fulfilled. First, individual-level *months until eHC eligibility* is exogenously determined by (i) the place of residence to which refugees have been allocated by authorities and (ii) the timing of the asylum decision. To the best of our knowledge, regional dispersal is quasi random [80] and follows the *Königsteiner Schlüssel*, which allocates refugees across federal states based on population size and tax revenues. Although fiscal capacity plays a role, this is equalized through Germany’s fiscal equalization scheme (German: *Länderfinanzausgleich*). Moreover, while some reception centers are ‘specialized’ by country of origin, we control for citizenship, and the largest group in our sample, Syrians (70% of our sample), are represented across all states [81]. Refugees have no influence over their assigned place of residence and cannot self-select into regions with earlier eHC access based on health, since health status is not part of the allocation algorithm.

A potential threat to the exclusion restriction would arise if regional characteristics that influence employment are correlated with early eHC implementation. We therefore compare regions with and without early eHC access along several relevant dimensions: unemployment rate [82], urbanity [83], immigrant enclaves presence [84], far-right AfD vote share (as a proxy for anti-immigrant sentiments) [82], and regional welfare generosity towards immigrants [85]. Supplementary Table S3 in S1 File shows that early eHC regions are somewhat more urban, have a higher unemployment rate and a lower overall foreigner share, though a higher share of refugees among foreigners. The AfD vote share is only marginally higher (+0.4 percentage points). Conditional on these covariates, it is unlikely that eHC access affects employment through channels other than health.

In Supplementary Tables S4 and S5 in S1 File, we verify that the 2^nd^-stage results of our main IV estimates stay qualitatively unchanged when including a set of additional district-level control variables, such as proxies for economic power and economic structure, demographic composition with regard to gender and age, childcare availability and quality as well as the housing market. Additionally, we performed a falsification exercise: Based on data from the Socio-Economic Panel [86] and IAB-SOEP Migration Sample [87,88] we compare the health status of the (non-refugee) migrant as well as native-born population across districts with and without eHC implementation. As depicted in Supplementary Tables S6, S7 and S8 in S1 File, we do not find any evidence in direction of a favorable health status of these populations in regions having implemented this refugee-specific reform.

It is also implausible that refugees can strategically influence the timing of the asylum decision. Most applicants submit their claims shortly after arrival and the duration of the asylum procedure depends on administrative processing beyond individual control. Even if such manipulation were possible, all applicants would have an incentive to speed up the process due to multiple benefits linked to approval – not just healthcare access, but also freedom of movement, work permit, family reunification right, and more generous social benefits. Taken together, both the assigned place of residence and asylum decision timing are exogenous to refugees, supporting the plausibility of the exclusion restriction for the first instrument.

Second, the regional *availability of medical services* is plausibly exogenous, since refugees are assigned to districts by the authorities and cannot choose their residence at this level (there are about 400 districts in Germany). Even if some attempt to relocate within a district to be closer to a doctor within the assigned district – this incentive may exist for all refugees, regardless of the waiting time-reducing reform – this does not affect our instrument, which varies at the district level. Thus, any such within-district movement does not undermine the validity of the instrument.

Third, *pre-migration satisfaction with health* is measured using retrospective self-reports of satisfaction with health collected when respondents participate in the survey for the first time. This measure refers to the time of the onset of the events that triggered migration, such as the war, or the conflict in respondents’ origin countries. While this baseline health measure may correlate with pre-migration educational [89] or employment histories [90], we explicitly control for these in our models. After conditioning on relevant covariates, we argue that pre-migration health status influences employment only via its effect on current health, thereby satisfying the exclusion restriction.

To assess the strength and validity of our instruments, we perform standard diagnostic tests as part of the IV estimation. Specifically, we report the first-stage Kleibergen-Paap rk Wald F-Statistic to test for weak instruments [91], the Kleibergen-Paap rk LM statistic to test for underidentification [91] and the Hansen J test of overidentifying restrictions to examine instrument exogeneity [92].

## 4. Results

### 4.1. OLS results

Table 2 presents OLS estimates of the association between health and employment (MCS: columns 1–3, PCS columns 4–6). Both MCS (column 1) and PCS (column 4) are positively and statistically significantly associated with employment probability in the pooled sample. A one standard deviation increase in MCS (PCS) corresponds to 1.8 (1.9) percentage points increase in the probability of being employed. Given an average employment rate of around 17 percent, this represents an increase of almost 9 percent.

**Table 2:** OLS Results: Employment.

*Outcome:*	1[Employed]					
*Sample:*	Pooled	Females	Males	Pooled	Females	Males
MCS	0.16***	0.04	0.28***			
	(0.04)	(0.04)	(0.06)			
PCS				0.19***	0.07	0.33***
				(0.05)	(0.04)	(0.08)
Person observations	3,454	1,376	2,078	3,454	1,376	2,078
Person-year observations	5,041	1,935	3,106	5,041	1,935	3,106
R2	0.206	0.144	0.206	0.206	0.145	0.206
Mean of dependent variable	0.166	0.048	0.240	0.166	0.048	0.240

Notes: Further included confounding variables described in section 3.4 are not reported. Coefficients and standard errors are multiplied by 100 for readability. Standard errors clustered at person-level in parentheses. * p < 0.10, ** p < 0.05, *** p < 0.01.

Source: IAB-BAMF-SOEP Survey of Refugees, v36.

Gender-specific results reveal that the association is driven by males: among men, both MCS and PCS are positively and significantly associated with employment (3.2 percentage points, or 7.5 percent increase), whereas the estimates for women are small and statistically insignificant.

### 4.2. IV Results

Supplementary Table S9 in S1 File provides the first-stage estimates of the 2SLS models and Table S10 in S1 File shows the reduced form. Supplementary Figure S1 in S1 File plots the predicted health values from the first-stage. Results suggest that longer waiting times until eHC eligibility reduce current health status among refugees who already reported poor pre-migration health – but only when treatment access is available (i.e., when the mean distance to a general practitioner is low). This supports the instruments’ relevance and heterogeneity in exposure.

Table 3 displays the second-stage 2SLS estimates. For both MCS and PCS, the IV coefficients are larger than their OLS counterparts. This may, first, be due to classical measurement error, which tends to bias the OLS coefficients toward zero. Second, IV estimates measure the local average treatment effect for compliers (those affected by the instrument), while OLS measures the average treatment effect for the entire population. If treatment effects are larger for compliers (i.e., refugees whose health status is affected only by changes in access to medical treatment), IV estimates may exceed their OLS counterparts.

**Table 3:** IV 2SLS results.

*Outcome:*	1[Employed]					
*Sample:*	Pooled	Females	Males	Pooled	Females	Males
MCS	0.62	0.50**	0.79			
	(0.42)	(0.21)	(0.73)			
PCS				0.43*	0.23	0.61
				(0.23)	(0.18)	(0.39)
Person observations	3,454	1,376	2,078	3,454	1,376	2,078
Person-year Observations	5,041	1,935	3,106	5,041	1,935	3,106
Mean of dependent variable	16.6	4.8	24.0	16.6	4.8	24.0
Underidentification: Kleibergen-Paap rk LM statistic	38.2	35.7	17.4	147.3	88.1	65.8
p-value Kleibergen-Paap rk LM	0.001	0.002	0.293	0.000	0.000	0.000
Weak identification: Kleibergen-Paap rk Wald F stat.	2.9	3.0	1.3	10.8	7.3	5.0
Overidentification: Hansen J statistic	13.2	27.0	12.2	12.8	28.8	10.0
p-value of Hansen J	0.508	0.019	0.590	0.542	0.011	0.761

Notes: Further included confounding variables described in section 3.4 are not reported. Coefficients and standard errors are multiplied by 100 for readability. Standard errors clustered at person-level in parentheses. * p < 0.10, ** p < 0.05, *** p < 0.01.

*Source*: IAB-BAMF-SOEP Survey of Refugees, v36.

For MCS, the pooled 2SLS estimate is positive (0.62) but statistically insignificant. Gender-disaggregated results show a large and statistically significant effect for women (0.50), but not for men. However, this finding should be interpreted with caution. The Kleibergen-Paap F-statistics in the MCS models are well below the common rule-of-thumb of 10, indicating weak instruments [93]. As such, the estimates – particularly for MCS – should be viewed as suggestive rather than conclusive evidence.

In contrast, instruments for PCS are stronger (F = 10.8 in pooled sample), lending more credibility to those estimates. The pooled 2SLS coefficient for PCS is 0.43 and statistically significant at the 10% level, confirming a positive effect of physical health on employment. Gender-specific estimates suggest stronger effects for men (0.61) than for women (0.23), although neither reaches conventional significance levels (p = 0.116 for men; p = 0.215 for women).

### 4.3. Mechanisms

In this section, we examine one plausible mechanism linking health to employment: refugees’ acquisition of host-country language skills. Language proficiency is widely recognized as a central facilitator of labor market integration in refugee-receiving countries. According to Ager and Strang’s [94] framework, language serves as a `facilitator’ of integration, enhancing both the transferability of pre-migration human capital [95] and the returns to post-migration investments in education and training [96]. If better mental or physical health improves language acquisition, this may constitute an indirect pathway through which health fosters employment participation.

We begin by analyzing the relationship between health status and German language proficiency. In the OLS models (Table 4, columns 1–6), MCS and PCS are both positively associated with self-reported language proficiency, but the estimated coefficients are small and only weakly significant. As in the case of employment, these results may suffer from endogeneity, especially reverse causality. Prior research suggests that limited language proficiency reduces health literacy [97] and complicates communication with medical professionals [98], which could impair self-reported health and create feedback loops.

**Table 4:** OLS & IV results: Language proficiency.

*Method:*	OLS						IV 2SLS					
*Sample:*	Pooled	Females	Males	Pooled	Females	Males	Pooled	Females	Males	Pooled	Females	Males
MCS	1.21***	1.12**	1.31***				11.62***	13.37***	−0.34			
	(0.32)	(0.53)	(0.38)				(3.69)	(3.86)	(5.06)			
PCS				2.00***	1.99***	1.93***				5.01***	8.43***	2.58
				(0.40)	(0.61)	(0.52)				(1.78)	(2.61)	(2.35)
Person observations	3,453	1,375	2,078	3,453	1,375	2,078	3,453	1,375	2,078	3,453	1,375	2,078
Person-year Observations	5,038	1,933	3,105	5,038	1,933	3,105	5,038	1,933	3,105	5,038	1,933	3,105
R2	0.393	0.354	0.400	0.395	0.356	0.401						
Mean of dependent variable	5.870	5.074	6.366	5.870	5.074	6.366	5.870	5.074	6.366	5.870	5.074	6.366
Underidentification: Kleibergen-Paap rk LM statistic							38.2	35.6	17.4	148.1	88.4	65.9
p-value Kleibergen-Paap rk LM							0.001	0.002	0.293	0.000	0.000	0.000
Weak identification: Kleibergen-Paap rk Wald F statistic							2.9	3.0	1.3	10.9	7.3	5.0
Overidentification: Hansen J statistic							16.4	17.0	21.0	25.3	25.8	19.7
p-value of Hansen J							0.291	0.257	0.102	0.032	0.027	0.138

Notes: Further included confounding variables described in section 3.4 are not reported. Coefficients and standard errors are multiplied by 100 for readability. Standard errors clustered at person-level in parentheses. * p < 0.10, ** p < 0.05, *** p < 0.01.

Source: IAB-BAMF-SOEP Survey of Refugees, v36.

The IV estimates (Table 4, columns 7–12) address this concern and reveal much stronger associations between health and language proficiency. Both MCS and PCS show large, statistically significant effects for the pooled and female samples, but no effect for male refugees. These results suggest that improvements in mental and physical health causally enhance German language skills among female but not male refugees. However, due to weak instruments – particularly for MCS – these findings should be treated as suggestive rather than conclusive.

What might explain gendered effects? One possible mechanism is access to formal language training. Table 5 presents OLS and IV estimates of the effect of health on language course participation. The IV estimates reveal that both MCS and PCS significantly increase the probability of participating in a language course – but only among female refugees. For men, neither health measure is significantly associated with course participation. Bearing in mind the weak instruments in the gender sub-samples, this suggests that better health may enable women to access formal training opportunities, which in turn improves their language skills.

**Table 5:** OLS & IV Results: Language Course Participation.

*Method:*	OLS						IV 2SLS					
*Sample:*	Pooled	Female	Male	Pooled	Female	Male	Pooled	Female	Male	Pooled	Female	Male
MCS	0.11**	0.22**	0.04				0.79	1.96***	−0.64			
	(0.05)	(0.09)	(0.05)				(0.60)	(0.70)	(0.71)			
PCS				−0.00	−0.03	0.03				0.50*	1.17**	0.02
				(0.06)	(0.11)	(0.07)				(0.30)	(0.52)	(0.34)
Person observations	3,438	1,371	2,067	3,438	1,371	2,067	3,438	1,371	2,067	3,438	1,371	2,067
Person-year Observations	4,992	1,920	3,072	4,992	1,920	3,072	4,992	1,920	3,072	4,992	1,920	3,072
R2	0.157	0.156	0.110	0.156	0.153	0.110						
Mean of dependent variable	0.811	0.696	0.883	0.811	0.696	0.883	0.811	0.696	0.883	0.811	0.696	0.883
Underidentification: Kleibergen-Paap rk LM statistic							38.4	36.2	17.0	148.9	89.5	66.9
p-value Kleibergen-Paap rk LM							0.001	0.002	0.319	0.000	0.000	0.000
Weak identification: Kleibergen-Paap rk Wald F statistic							2.9	3.0	1.3	10.9	7.4	5.2
Overidentification: Hansen J statistic							15.7	17.5	6.0	14.7	23.6	7.3
p-value of Hansen J							0.331	0.231	0.967	0.397	0.052	0.922

Notes: Further included confounding variables described in section 3.4 are not reported. Coefficients and standard errors are multiplied by 100 for readability. Standard errors clustered at person-level in parentheses. * p < 0.10, ** p < 0.05, *** p < 0.01.

Source: IAB-BAMF-SOEP Survey of Refugees, v36.

Importantly, language proficiency is often a precondition for occupational entry – especially in highly regulated sectors [99]. Many refugee women with pre-migration work experience were employed in the health or education sectors, where access to jobs in Germany typically requires formal recognition of qualifications and advanced language skills [17]. In contrast, male refugees are more likely to work in less regulated, lower-threshold sectors such as logistics or construction, where immediate employment is possible even with limited language proficiency. These sectoral differences in occupational requirements likely contribute to our observed suggestive gendered patterns in the health-language-employment nexus.

## 5. Discussion and conclusion

Public and media discourses often portray refugees as highly vulnerable individuals with complex health needs and limited labor market potential [100–102]. In these narratives, healthcare expenditures on refugees are seen more as a fiscal burden than a societal investment. At the same time, descriptive evidence suggests a link between health and employment among refugee populations, though the direction and strength of this relationship remain unclear. Against this backdrop, our study posed a straightforward yet underexplored causal question: Does better health improve refugees’ chances of employment? Specifically, we examined whether mental and physical health causally influence labor market entry, leveraging a natural experiment based on refugees’ quasi-exogenous dispersal across German districts and variation in healthcare access.

Our findings yield three key insights. First, both mental and physical health are positively associated with employment. Second, 2SLS estimates indicate a positive causal effect of physical health on refugees’ employment. Mental health shows a positive causal effect only for female refugees’ employment – although this must be interpreted cautiously due to weak instruments. Third, our mechanism analysis shows that better health facilitates German language acquisition. Suggestive evidence shows that this holds specifically for women and is driven by increasing participation in formal language courses.

Gendered effects may reflect differences in pre-migration occupational profiles. While we do not trace continuity across sectors, previous research shows that refugee women were more likely than men to have worked in highly regulated fields such as healthcare or education prior to arrival (Kosyakova et al. [17]). In Germany, re-entering such sectors typically requires formal credential recognition and advanced language proficiency. By contrast, men more often find work in less regulated, lower-threshold sectors such as logistics or construction, where physical fitness can be more decisive than language skills.

Our findings thus extend earlier research that emphasizes the role of language as a ‘facilitator’ of integration (Ager and Strang [94]). For women, better health not only increases participation in language courses, but also improves language proficiency – a likely pathway towards employment. In contrast, male refugees, who often enter the labor market more quickly, may acquire German on the job. This gender-specific mechanism highlights the importance of connecting the dots from health to language acquisition, and from language acquisition to employment, particularly for refugee women.

From a policy perspective, our results suggest that improving access to healthcare is not only an issue of equity but also a tool for economic inclusion. Timely and comprehensive medical support can yield indirect economic returns by improving refugees’ employability, especially when paired with language and vocational training. For social workers and integration practitioners, the results highlight the value of bundling health support with employment and language services. For employers, understanding refugees’ health-related vulnerabilities may help improve workplace support and retention. And for refugees themselves, investing in health – by utilizing available services – can be a vital step toward long-term integration.

Our findings become particularly relevant in light of recent policy developments in Germany. Since early 2024, the waiting period for access to regular healthcare under the Asylum Seekers’ Benefits Act (AsylbLG) has been extended to 36 months, considerably prolonging restricted healthcare access for asylum seekers [49]. Furthermore, Ukrainian refugees arriving since April 2025 are subject to the AsylbLG and thus face similar limitations in accessing the healthcare system [103]. Our results suggest that such prolonged restrictions may not only affect refugees’ health but could also delay labor market integration by limiting timely access to necessary medical treatment.

At the same time, the study has limitations. Our causal interpretation rests on the standard assumptions of the instrumental variable approach. First, although the exclusion restriction appears plausible based on the institutional context and the robustness checks presented in the paper, it cannot be formally tested [104]. Second, instrument relevance is limited in some specifications. In particular, the instruments used to identify effects of mental health on employment do not reach the common rule-of-thumb for the first-stage F-statistic (>10), indicating potential weak-instrument concerns. For the instrument of waiting time until eligibility for comprehensive healthcare, this could reflect the limited availability of mental health services in Germany, particularly psychotherapy [105]. Access to such services may be especially constrained for refugees due to language barriers and the complexity of trauma-related care needs [106]. Consequently, the IV estimates related to mental health should be interpreted cautiously and viewed as suggestive rather than conclusive evidence.

Further research should build on these findings by examining other integration dimensions, such as job quality, upward mobility or employment sustainability, and by identifying additional mechanisms beyond language acquisition. Further efforts to improve measurement and causal identification, especially in the domain of mental health, would substantially enhance the evidence base.

## Supporting information

S1 File Additional Figures and Tables. (PDF)
