# Supplementary material for: The effect of health on refugees’ labor market integration: Evidence from a natural experiment in Germany
Source: PLoS One. 2026 Apr 20;21(4):e0346936. doi: 10.1371/journal.pone.0346936 (PMC13094989; doi:10.1371/journal.pone.0346936)
Supplement: S1 File — (PDF) [file pone.0346936.s001.pdf]

The effect of health on refugees' labor market integration: Evidence from a natural experiment in Germany

Additional Figures and Tables

Table S1: Regional coverage and implementation timeline of eHCs

Federal state	District	Municipality	Month of implementation
Baden-Wuerttemberg	-	-	-
Bavaria	-	-	-
Berlin	All	All	January 2016
Brandenburg	Landkreis Teltow-Fläming	All	September 2016
	Oberhavel	All	October 2016
	Potsdam	All	July 2016
	Dahme-Spreewald	All	January 2017
	Havelland	All	January 2017
	Potsdam-Mittelmark	All	January 2017
	Cottbus	All	January 2017
	Barnim	All	February 2017
	Uckermark	All	February 2017
	Frankfurt Oder	All	February 2017
	Oder-Spree	All	April 2017
	Prignitz	All	April 2017
	Brandenburg an der Havel	All	April 2017
	Spree-Neiße	All	January 2018
	Elbe-Elster	All	October 2017
	Oberspreewald-Lausitz	All	October 2017
	Ostprignitz-Ruppin	All	January 2019
Bremen	All	All	January 2015
Hamburg	All	All	January 2012
Hesse	-	-	-
Lower Saxony	Delmenhorst	All	January 2017
		Cuxhaven	January 2019
		Burgwedel	January 2018
Mecklenburg-Hither Pomerania	-	-	-
North Rhine-Westphalia	Bonn	All	January 2016
	Bochum	All	January 2016
	Mülheim an der Ruhr	All	January 2016
	Köln	All	March 2016
	Münster	All	March 2016
	Düsseldorf	All	April 2016
	Oberhausen	All	April 2016
	Remscheid	All	April 2016
	Mönchengladbach	All	July 2016
		Gevelsberg	January 2016
		Monheim am Rhein	January 2016
		Wetter (Ruhr)	March 2016
		Herdecke	April 2016
		Dülmen	April 2016
		Hattingen	April 2016
		Alsdorf	March 2016
		Leichlingen (Rheinland)	January 2016
		Wermelskirchen	February 2016
		Bocholt	March 2016
		Moers	April 2016
		Sprockhövel	Aril 2016
		Gladbeck	January 2017
		Hennef	January 2017
		Troisdorf	January 2017
		Bornheim	January 2017
		Neukirchen-Vluyn	January 2019
		Recklinghausen	January 2019
		St. Augustin	January 2018
Rheineland-Palatinate	Trier	All	January 2017
	Mainz	All	July 2017
	Kusel	All	July 2017
Saarland	-	-	-
Saxony	-	-	-
Saxony-Anhalt	-	-	-
Schleswig-Holstein	All	All	January 2016
Thuringia	All	All	January 2017

Table S2: Detailed summary statistics

Variable	Mean	SD	Min	Max	Median	N
Female	0.38		0	1		5,041
At least 1 child lives in household	0.67		0	1		5,041
Age: 18 - 25	0.27		0	1		5,041
26-35	0.34		0	1		5,041
36-45	0.24		0	1		5,041
> 45	0.15		0	1		5,041
Partnership status: Single	0.28		0	1		5,021
Partner lives in Germany	0.64		0	1		5,021
Partner lives abroad	0.09		0	1		5,021
Education before immigration (ISCED-A): 0	0.09		0	1		4,872
ISCED-A: 1 (primary education)	0.15		0	1		4,872
ISCED-A: 2 (Lower secondary)	0.29		0	1		4,872
ISCED-A: 3/4 (Upper secondary, non-tertiary)	0.26		0	1		4,872
ISCED-A: 5/6/7/8 (Tertiary education)	0.21		0	1		4,872
Country of origin illiterate	0.05		0	1		5,038
Was employed before migration	0.66		0	1		4,979
Citizenship: Syria	0.69		0	1		5,041
Afghanistan	0.08		0	1		5,041
Iraq	0.12		0	1		5,041
Eritrea	0.05		0	1		5,041
MENA	0.03		0	1		5,041
Western Balkans	0.01		0	1		5,041
Former USSR	0.00		0	1		5,041
Rest Africa	0.02		0	1		5,041
Other or stateless	0.00		0	1		5,041
No too severe traumatic experience during migration	0.40		0	1		5,041
Severe traumatic exp. during migration (sexual harassment, physical assault, shipwreck, robbery, prison)	0.24		0	1		5,041
Refuses to report	0.36		0	1		5,041
German score before immigration (1 bad - 5 good)	1.1	0.4	1	5	1.0	5,033
<= 1 year since arrival	0.08		0	1		5,041
1 < years since arrival <= 2	0.34		0	1		5,041
2 < years since arrival <= 3	0.23		0	1		5,041
3 < years since arrival <= 4	0.25		0	1		5,041
> 4 years since arrival	0.09		0	1		5,041
Months between arrival and asylum approval	10.7	7.6	0	69	9	5,041
Lives in private rather than communal accommodation	0.79		0	1		5,018
Never been discriminated based on origin	0.65		0	1		4,982
Seldom	0.29		0	1		4,982
Often	0.05		0	1		4,982
No worries about prospects of staying in Germany	0.46		0	1		5,001
Some worries	0.26		0	1		5,001
High worries	0.28		0	1		5,001
Felt welcome in Germany at arrival (1 not at all - 5 strongly)	4.6	0.8	1	5	5	5,010
Unemployment rate [%] (January of arrival year)	7.2	2.9	1.5	16.9	6.8	5,041
Population density [per sqkm] (December of arrival year - 1)	924	1077	38	4682	366	5,041
Foreigner share in population [%] (December of arrival year - 1)	9.3	5.0	0.9	28.0	8.5	5,041
Refugee share in foreigners [%] (December of arrival year - 1)	14.8	12.2	2.0	103.4	12.0	5,041
AfD voting share [%] (federal election 2013)	4.6	1.0	2.2	7.9	4.6	5,011

Table S3: Balance table between districts with and without eHC

Variable	No eHC	eHC	Difference
Unemployment rate [%]	6.048	8.261	2.213***
	(2.734)	(2.580)	(0.376)
Population density (per sqkm)	496.561	719.355	222.794**
	(651.564)	(871.121)	(97.564)
Foreigner share [%]	7.559	5.610	-1.949***
	(4.484)	(4.101)	(0.613)
Refugee in foreigners share [%]	12.042	15.395	3.353***
	(9.300)	(6.359)	(1.222)
AfD voting share	4.561	4.943	0.382**
	(1.034)	(1.170)	(0.148)
Observations	253	65	318

Notes: Standard deviation in parentheses. Analysis at the district level. Sample includes 318 of the 401 total districts in Germany to which at least one refugee in our sample has been dispersed. * $p<0.10$, ** $p<0.05$, *** $p<0.01$.

Table S4: Robustness – 2nd stage MCS results with more district-level controls

Outcome:	**1[Employed]**									
	(1)	(2)	(3)	(4)	(5)	(6)	(7)	(8)	(9)	(10)
MCS	0,65	0,61	0,65	0,67	0,63	0,63	0,63	0,68	0,62	0,67*
	(0,42)	(0,42)	(0,42)	(0,42)	(0,42)	(0,42)	(0,42)	(0,42)	(0,42)	(0,40)
GDP p.c.	0,08									0,16
	(0,05)									(0,10)
Tax revenue		0,00								-0,01
		(0,00)								(0,01)
Employees in service sector (%)			0,10							0,00
			(0,06)							(0,08)
Firms with < 10 employees (%)				-0,27						0,35
				(0,29)						(0,45)
Mean age of the population					-1,23**					-1,12**
					(0,48)					(0,53)
Women (%)						0,51				0,23
						(0,99)				(1,14)
Childcare coverage (<3 years)							0,05			0,01
							(0,07)			(0,09)
Number of childcare places per pedagogical staff								-0,26		-0,21
								(0,36)		(0,39)
Rental prices									0,70	0,19
									(0,51)	(0,78)
Person observations	3.454	3.454	3.454	3.454	3.454	3.454	3.454	3.454	3.454	3.454
Person-year Observations	5.041	5.041	5.041	5.041	5.041	5.041	5.041	5.041	5.041	5.041
R2	0,187	0,190	0,187	0,185	0,189	0,188	0,188	0,184	0,189	0,187
Mean of dependent variable	0,166	0,166	0,166	0,166	0,166	0,166	0,166	0,166	0,166	0,166
Underidentification: Kleibergen-Paap rk LM statistic	38,8	38,3	38,8	38,9	39,0	38,2	38,2	38,2	38,3	42,5
p-value Kleibergen-Paap rk LM	0,001	0,001	0,001	0,001	0,001	0,001	0,001	0,001	0,001	0,000
Weak identification: Kleibergen-Paap rk Wald F statistic	2,9	2,9	2,9	3,0	3,0	2,9	2,9	2,9	2,9	3,2
Overidentification: Hansen J statistic	11,9	13,2	12,5	12,4	12,2	12,8	13,5	12,6	13,1	10,8
p-value of Hansen J	0,614	0,511	0,567	0,573	0,593	0,541	0,490	0,560	0,517	0,704

Notes: Remaining control variables correspond to the setup of Table 3 in the manuscript. Coefficients and standard errors are multiplied by 100 for readability. Standard errors clustered at person-level in parentheses. * p<0.10, ** p<0.05, *** p<0.01.

Table S5: Robustness – 2nd stage PCS results with more district-level controls

Outcome:	1[Employed]									
	(1)	(2)	(3)	(4)	(5)	(6)	(7)	(8)	(9)	(10)
PCS	0,42*	0,43*	0,44*	0,42*	0,44*	0,45*	0,43*	0,46**	0,43*	0,46*
	(0,24)	(0,23)	(0,23)	(0,23)	(0,23)	(0,24)	(0,24)	(0,24)	(0,23)	(0,24)
GDP p.c.	0,05									0,09
	(0,05)									(0,10)
Tax revenue		0,00								-0,00
		(0,00)								(0,01)
Employees in service sector (%)			0,08							-0,01
			(0,06)							(0,08)
Firms with < 10 employees (%)				-0,12						0,36
				(0,28)						(0,44)
Mean age of the population					-1,04**					-1,08**
					(0,46)					(0,53)
Women (%)						0,61				0,59
						(0,99)				(1,13)
Childcare coverage (<3 years)							0,05			0,04
							(0,07)			(0,08)
Number of childcare places per pedagogical staff								-0,28		-0,23
								(0,37)		(0,40)
Rental prices									0,53	-0,09
									(0,51)	(0,77)
Person observations	3.454	3.454	3.454	3.454	3.454	3.454	3.454	3.454	3.454	3.454
Person-year Observations	5.041	5.041	5.041	5.041	5.041	5.041	5.041	5.041	5.041	5.041
R2	0,203	0,203	0,203	0,203	0,203	0,202	0,203	0,202	0,203	0,203
Mean of dependent variable	0,166	0,166	0,166	0,166	0,166	0,166	0,166	0,166	0,166	0,166
Underidentification: Kleibergen-Paap rk LM statistic	145,2	146,3	147,8	147,7	147,4	147,3	145,6	145,5	147,2	143,3
p-value Kleibergen-Paap rk LM	0,000	0,000	0,000	0,000	0,000	0,000	0,000	0,000	0,000	0,000
Weak identification: Kleibergen-Paap rk Wald F statistic	10,7	10,7	10,8	10,8	10,8	10,7	10,7	10,7	10,8	10,5
Overidentification: Hansen J statistic	12,2	12,8	12,1	12,7	11,8	12,3	13,1	12,1	12,8	11,0
p-value of Hansen J	0,591	0,545	0,597	0,553	0,623	0,586	0,518	0,595	0,543	0,684

Notes: Remaining control variables correspond to the setup of Table 3 in the manuscript. Coefficients and standard errors are multiplied by 100 for readability. Standard errors clustered at person-level in parentheses. * p<0.10, ** p<0.05, *** p<0.01.

Table S6: Mean MCS and PCS scores of native-born and non-refugee migrants

	Native-born		Non-refugee migrants	
	Regions without eHC	Regions with eHC	Regions without eHC	Regions with eHC
MCS	51.23	51.06	51.77	50.83
	(9.809)	(10.066)	(9.329)	(9.548)
PCS	49.28	48.90	49.98	50.61
	(10.019)	(10.291)	(10.118)	(9.925)
Observations	30,799	9,502	6,184	1,772

Notes: Table reports the mean of MCS and PCS with standard deviation in brackets. Natives are defined as individuals born in Germany. Non-refugee migrants are defined as individuals born abroad, without a refugee background.
Sources: SOEP (2021), IAB-SOEP (2021).

Table S7: Multivariate comparison of MCS & PCS scores among native-born

	MCS				PCS			
	(1)	(2)	(3)	(4)	(5)	(6)	(7)	(8)
Region with eHCs	-0.172	-0.172	-0.234*	-0.262*	-0.395***	-0.395***	-0.138	-0.298**
	(0.138)	(0.138)	(0.136)	(0.136)	(0.148)	(0.148)	(0.129)	(0.127)
Gender		-2.156***	-2.123***	-2.049***		-0.959***	-1.100***	-0.739***
(female==1)		(0.115)	(0.114)	(0.115)		(0.124)	(0.109)	(0.107)
Age			0.061***	0.056***			-0.252***	-0.266***
			(0.003)	(0.003)			(0.003)	(0.003)
Education								
(ref: ISCED-0)								
ISCED-1				-0.837				-1.223*
				(1.034)				(0.661)
ISCED-2				0.045				-1.622***
				(0.991)				(0.624)
ISCED-3/4				0.373				0.607
				(0.977)				(0.607)
ISCED-5-8				1.024				3.471***
				(0.980)				(0.610)
Observations	39,655	39,655	39,655	39,655	39,655	39,655	39,655	39,655
R2	0.000	0.012	0.024	0.026	0.000	0.003	0.205	0.230

Notes: OLS estimates for a sample including only native individuals, defined as individuals born in Germany. Standard errors are clustered at the individual level, in parenthesis. * p<0.10, ** p<0.05, *** p<0.01.
Sources: SOEP (2021).

Table S8: Multivariate comparison of MCS & PCS scores among non-refugee migrants

	MCS				PCS			
	(1)	(2)	(3)	(4)	(5)	(6)	(7)	(8)
Region with eHCs	-.912***<br>(.294)	-.940***<br>(.292)	-.949***<br>(.292)	-.929***<br>(.292)	.657**<br>(.321)	.640**<br>(.321)	.438<br>(.287)	.296<br>(.282)
Gender<br>*(female==1)*		-1.803***<br>(.240)	-1.825***<br>(.240)	-1.798***<br>(.240)		-1.144***<br>(.274)	-1.637***<br>(.245)	-1.660***<br>(.241)
Age			-0.013<br>(.009)	-.015*<br>(.009)			-.298***<br>(.009)	-.299***<br>(.008)
Education<br>*(ref: ISCED-0)*								
ISCED-1				2.193<br>(2.450)				1.836<br>(2.645)
ISCED-2				4.478*<br>(2.428)				2.596<br>(2.614)
ISCED-3/4				4.870*<br>(2.413)				4.211<br>(2.601)
ISCED-5-8				5.017*<br>(2.415)				6.586<br>(2.602)
Observations	7,891	7,891	7,891	7,891	7,891	7,891	7,891	7,891
R2	0.002	0.011	0.011	0.016	0.001	0.004	0.178	0.201

Notes: OLS estimates for sample including only non-refugee migrants, defined as individuals born outside of Germany without a refugee background. Standard errors are clustered at the individual level, in parenthesis. * p<0.10, ** p<0.05, *** p<0.01.

Source: IAB-SOEP (2021).

Table S9: IV first-stage results

Outcome:	MCS			PCS		
Sample:	**Both**	**Females**	**Males**	**Both**	**Females**	**Males**
	(1)	(2)	(3)	(4)	(5)	(6)
Months until eHC eligibility: 1 - 8 (ref: 0)	108.6	195.1	-336.4	-184.5	3.0	-719.3
	(352.2)	(612.5)	(467.4)	(372.5)	(439.4)	(947.7)
9 - 14	-328.3	393.7	-1034.6*	-259.6	264.9	-707.9
	(406.5)	(653.7)	(551.0)	(394.1)	(446.4)	(962.4)
15	-544.0	-508.7	-822.4	-474.5	536.6	-1390.2
	(389.7)	(643.9)	(509.0)	(410.7)	(446.8)	(990.8)
Satisfaction with health before migration	1.6	53.0	-65.7	68.1*	140.6***	-5.0
	(36.9)	(62.4)	(47.1)	(38.5)	(39.6)	(100.8)
Months until eHC eligibility: 1 - 8 # Satisfaction with health before migration	-7.1	-11.6	42.0	15.1	-16.3	79.1
	(41.2)	(71.4)	(52.1)	(43.0)	(52.4)	(103.3)
9 - 14 # Satisfaction with health before migration	33.7	-65.4	123.2**	25.2	-43.1	79.9
	(46.8)	(76.1)	(61.1)	(45.3)	(52.7)	(104.9)
15 # Satisfaction with health before migration	67.1	43.4	113.2*	49.0	-70.8	151.8
	(45.6)	(75.2)	(58.1)	(47.5)	(54.4)	(108.1)
GP distance	-26.5	14.4	-119.6	-56.6	-12.1	-139.0
	(40.4)	(46.1)	(78.7)	(42.2)	(35.3)	(193.2)
Months until eHC eligibility: 1 - 8 # GP distance	-3.4	-14.1	76.5	67.5	47.8	148.0
	(42.1)	(49.9)	(80.0)	(44.3)	(41.2)	(193.8)
9 - 14 # GP distance	10.3	-87.9	132.3	73.1	13.0	159.1
	(47.4)	(60.8)	(83.1)	(47.9)	(45.0)	(194.7)
15 # GP distance	52.1	5.4	149.6*	89.4*	23.5	190.1
	(45.7)	(58.4)	(82.3)	(47.5)	(45.9)	(195.5)
Satisfaction with health before migration # GP distance	4.4	-0.3	14.5*	6.6	1.2	15.9
	(4.6)	(5.7)	(8.1)	(4.9)	(4.5)	(20.7)
Months until eHC eligibility: 1 - 8 # Satisfaction with health before migration # GP distance	-0.9	0.2	-9.5	-7.1	-3.6	-16.7
	(4.9)	(6.2)	(8.4)	(5.1)	(5.1)	(20.8)
9 - 14 # Satisfaction with health before migration # GP distance	-2.0	10.5	-16.1*	-8.1	-0.2	-18.1
	(5.4)	(7.3)	(8.7)	(5.5)	(5.5)	(20.9)
15 # Satisfaction with health before migration # GP distance	-7.6	-0.2	-19.7**	-10.2*	-1.4	-21.9
	(5.4)	(7.2)	(8.9)	(5.5)	(5.8)	(21.0)
Person observations	3,454	1,376	2,078	3,454	1,376	2,078
Person-year Observations	5,041	1,935	3,106	5,041	1,935	3,106
R2	0.112	0.152	0.092	0.238	0.262	0.202
Mean of dependent variable	48.8	47.1	49.9	53.5	51.1	55.0

Notes: Further included confounding variables (not reported): Months between arrival and asylum approval; Age: (i) 18-25, (ii) 26-35; (iii) 36-45; (iv) > 45; Female; At least 1 child in household; Female X Child; Partnership: (i) single, (ii) lives in Germany, (iii) lives abroad); Education before immigration: (i) ISCED1, (ii) ISCED2, (iii) ISCED 3, 4, (iv) ISCED 5, 6, 7, 8; Employed before migration; Citizenship: (i) Syria, (ii) Afghanistan, (iii) Iraq, (iv) Eritrea, (v) MENA, (vi) Western Balkans, (vii) Former USSR, (viii) Rest Africa, (ix) Other or stateless; Years since arrival (5 cat.); Traumatic experience during escape: (i) no, (ii) yes, (iii) refuses to report; Survey year dummies; German score before immigration; Country of origin illiterate; Private vs. communal accommodation; Discrimination experience based on origin: (i) never, (ii) seldom, (iii) often; Worries about prospects of staying in Germany: (i) no, (ii) some, (iii) big; Degree person felt welcome in Germany at arrival; District-level variables, assigned district in arrival year – 1: (i) Unemployment rate, (ii) Population density, (iii) Foreigner share, (iv) Refugee share among foreigners, AfD federal election 2013 voting share. Supplementary Figure A1 shows predicted margins based on models (1) and (4). Coefficients and standard errors are multiplied by 100 for readability. Standard errors clustered at person-level in parentheses. * p<0.10, ** p<0.05, *** p<0.01.

Table S10: Reduced form results

Outcome:	1[Employed]		
Sample:	**Pooled**	**Females**	**Males**
	(1)	(2)	(3)
Months until eHC eligibility: 1 - 8 (ref: 0)	8,87	-2,18	10,41
	(8,25)	(11,25)	(18,87)
9 - 14	12,23	-9,75	12,92
	(8,96)	(10,56)	(20,31)
15	10,18	-15,35	16,28
	(10,01)	(10,50)	(21,34)
Satisfaction with health before migration	1,28	0,29	0,72
	(0,88)	(1,17)	(2,11)
Months until eHC eligibility: 1 - 8 # Satisfaction with health before migration	-0,84	-0,47	-0,37
	(1,03)	(1,31)	(2,22)
9 - 14 # Satisfaction with health before migration	-1,13	0,01	-0,34
	(1,11)	(1,27)	(2,39)
15 # Satisfaction with health before migration	-1,44	0,28	-1,33
	(1,23)	(1,22)	(2,50)
GP distance	1,40*	0,09	0,91
	(0,78)	(0,52)	(3,79)
Months until eHC eligibility: 1 - 8 # GP distance	-1,59*	-0,48	-1,31
	(0,85)	(0,62)	(3,82)
9 - 14 # GP distance	-1,89**	-0,10	-1,61
	(0,92)	(0,61)	(3,86)
15 # GP distance	-2,05**	-0,10	-2,15
	(1,00)	(0,58)	(3,92)
Satisfaction with health before migration # GP distance	-0,18*	-0,08	-0,08
	(0,10)	(0,06)	(0,42)
Months until eHC eligibility: 1 - 8 # Satisfaction with health before migration # GP distance	0,18	0,11	0,10
	(0,11)	(0,08)	(0,43)
9 - 14 # Satisfaction with health before migration # GP distance	0,20*	0,10	0,10
	(0,12)	(0,08)	(0,43)
15 # Satisfaction with health before migration # GP distance	0,25**	0,07	0,22
	(0,13)	(0,07)	(0,44)
Person observations	3.454	1.376	2.078
Person-year Observations	5.041	1.935	3.106
R2	0,206	0,158	0,204
Mean of dependent variable	0,166	0,048	0,240

Notes: Further included confounding variables (not reported): Months between arrival and asylum approval; Age: (i) 18-25, (ii) 26-35; (iii) 36-45; (iv) > 45; Female; At least 1 child in household; Female X Child; Partnership: (i) single, (ii) lives in Germany, (iii) lives abroad); Education before immigration: (i) ISCED1, (ii) ISCED2, (iii) ISCED 3, 4, (iv) ISCED 5, 6, 7, 8; Employed before migration; Citizenship: (i) Syria, (ii) Afghanistan, (iii) Iraq, (iv) Eritrea, (v) MENA, (vi) Western Balkans, (vii) Former USSR, (viii) Rest Africa, (ix) Other or stateless; Years since arrival (5 cat.); Traumatic experience during escape: (i) no, (ii) yes, (iii) refuses to report; Survey year dummies; German score before immigration; Country of origin illiterate; Private vs. communal accommodation; Discrimination experience based on origin: (i) never, (ii) seldom, (iii) often; Worries about prospects of staying in Germany: (i) no, (ii) some, (iii) big; Degree person felt welcome in Germany at arrival; District-level variables, assigned district in arrival year – 1: (i) Unemployment rate, (ii) Population density, (iii) Foreigner share, (iv) Refugee share among foreigners, AfD federal election 2013 voting share. Coefficients and standard errors are multiplied by 100 for readability. Standard errors clustered at person-level in parentheses. * p<0.10, ** p<0.05, *** p<0.01.

Figure S1: Predicted MCS & PCS

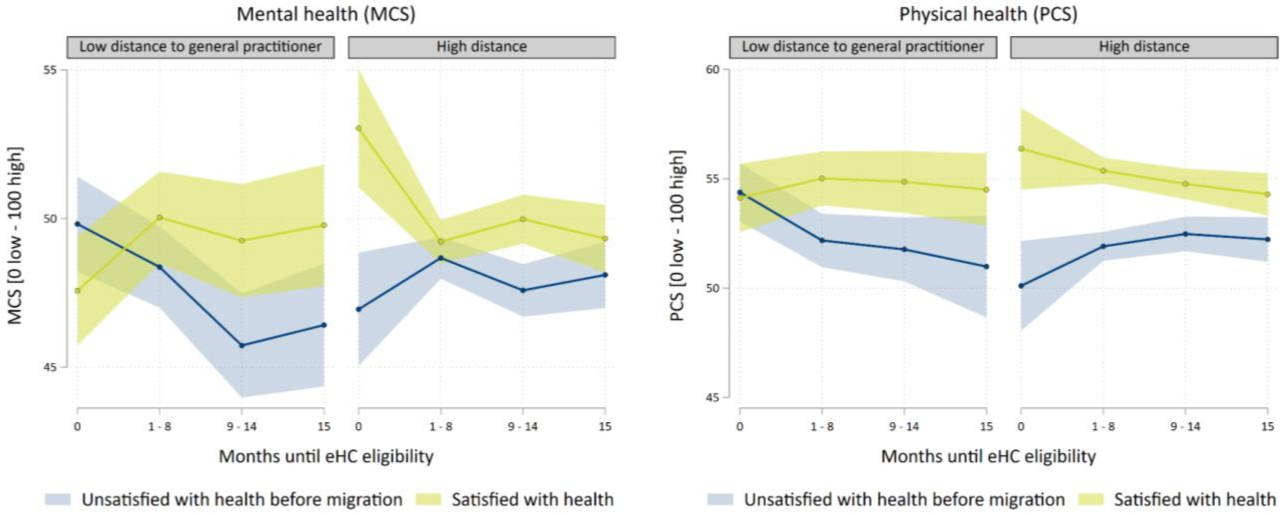

Notes: Figure shows predictive margins based on models 1 and 4 of supplementary Table A3. Included control variables: Months between arrival and asylum approval; Age: (i) 18-25, (ii) 26-35; (iii) 36-45; (iv) > 45; Female; At least 1 child in household; Female X Child; Partnership: (i) single, (ii) lives in Germany, (iii) lives abroad); Education before immigration: (i) ISCED1, (ii) ISCED2, (iii) ISCED 3, 4, (iv) ISCED 5, 6, 7, 8; Employed before migration; Citizenship: (i) Syria, (ii) Afghanistan, (iii) Iraq, (iv) Eritrea, (v) MENA, (vi) Western Balkans, (vii) Former USSR, (viii) Rest Africa, (ix) Other or stateless; Years since arrival (5 cat.); Traumatic experience during escape: (i) no, (ii) yes, (iii) refuses to report; Survey year dummies; German score before immigration; Country of origin illiterate; Private vs. communal accommodation; Discrimination experience based on origin: (i) never, (ii) seldom, (iii) often; Worries about prospects of staying in Germany: (i) no, (ii) some, (iii) big; Degree person felt welcome in Germany at arrival; District-level variables, assigned district in arrival year – 1: (i) Unemployment rate, (ii) Population density, (iii) Foreigner share, (iv) Refugee share among foreigners, AfD federal election 2013 voting share. Standard errors clustered at person-level. Shared areas denote 90 confidence intervals
